# DNA Methylation Activates TP73 Expression in Hepatocellular Carcinoma and Gastrointestinal Cancer

**DOI:** 10.1038/s41598-019-55945-7

**Published:** 2019-12-18

**Authors:** Zhixing Yao, Cristina Di Poto, Grace Mavodza, Everett Oliver, Habtom W. Ressom, Zaki A. Sherif

**Affiliations:** 1 Department of Biochemistry & Molecular Biology, College of Medicine, Howard University, Washington, DC 20059 USA; 2 Department of Oncology, Lombardi Cancer Center, Georgetown University, Washington, DC 20007 USA; 3 Department of Pharmacology, Hershey College of Medicine, Pennsylvania State University, Pennsylvania, PA 17033 USA

**Keywords:** Hepatocellular carcinoma, Hepatocellular carcinoma

## Abstract

The complexity of TP73 expression and its functionality, as well as the role of TP73 in tumorigenesis, unlike its cousin TP53, which is an established tumor suppressor, have remained elusive to date. In this study, we isolated two stem cell lines (HepCY & HepCO) from normal young and old human liver tissues. We determined TP73 expression in HepCY and HepCO, hepatocellular cancer (HCC) cell lines (HepG2, SNU398, SNU449 and SNU475), gastrointestinal cancer (GI) cell lines (Caco2 and HCT116) and normal skin fibroblasts cell line (HS27). Immunohistochemical analyses of TP73 expression was also performed in non-cancerous and adjacent cancerous liver tissues of HCC patients. The results show that TP73 expression is exclusive to the cancer cell lines and not the adjacent normal liver tissues. Moreover, methylation-specific PCR and bisulfite sequencing studies revealed that TP73 promoter is activated only in cancer cell lines by DNA methylation. Furthermore, ChIP assay results demonstrated that a chromosomal networking protein (CTCF) and tumor protein p53 (TP53) bind to TP73 promoter and regulate TP73 expression. Our observations demonstrate that a positive correlation in tumorigenesis exists between TP73 expression and DNA methylation in promoter regions of TP73. These findings may prove significant for the development of future diagnostic and therapeutic applications.

## Introduction

Mammalian *TP73* (p73) is a member of a gene family that comprises *TP63* (p63) and the well-characterized tumor suppressor *TP53* (p53). The broad range of functions regulated and generally controlled by these family members includes stem cells biology, cell fate, embryonic development, differentiation, reproduction, metabolic processes, genomic repair, senescence, and changes in epigenetic marks and tumor suppression^1^. But unlike p53, both p73 and p63, play pivotal roles in the normal development of mice^2^. However, in contrast to *TP53*, which is mutated in half of all human cancers, *TP63* and *TP73* are seldom mutated even though they are also involved in tumor suppression. There are structural and functional similarities among the three homologous proteins. As transcription factors, their activities are governed by unique and shared post-transcriptional modifications and regulatory cofactors. TP53 enhances cellular responses to stress and development; whereas p63 and p73 proteins play important roles in embryonic development and differentiation although their biological function is intricate. The *TP63* and *TP73* genes are transcribed into different isoforms that encode proteins with adversarial properties: the TA-isoforms exhibit tumor-suppressor activity and the DN-isoforms operate as proto-oncogenes^1^. The *TP73* gene encodes two different proteins, TAp73 (i.e. V1) and ΔNp73 (i.e. V2), and maps to the small arm of chromosome 1 (1p36), a region that is often deleted in several tumors and may harbor multiple tumor suppressor genes^3,4^. The current available data indicate that the major isoform and the full-length of the protein, TAp73α, is detectable in physiological systems^5,6^. As a transcription factor, p73 is activated in a similar manner to p53 in response to DNA damage and regulates the expression of downstream genes involved in cell cycle arrest and apoptosis^7–10^. However, there are other compounding functions of this gene that reflect its non-tumor-related characters, thus making it very difficult to assess its specific role in tumorigenesis^10–15^. In general, the p53 family performs as a signaling “network” engaging in crosstalk with various metabolic and stress signals to control cell development, differentiation, proliferation and death.

Epigenetic events that cause changes in gene expression are common in human cancers. These changes include DNA methylation, histone modifiers, microRNAs and chromatin remodelers^16^. Focal DNA hypermethylation of promoters of genes that are involved in tumor suppression and global hypomethylation of non-coding regions are both associated with gene-silencing in cancer^11,17^. DNA methylation and chromatin dysregulation can induce transcriptional repression at transcription start sites, which suggests their critical roles in tumorigenesis^18–20^.

CTCF is zinc finger protein that operates as a chromosomal networking protein CCCTC binding factor. This nuclear protein regulates and represses a wide range of genes including IGF2^21^. As a transcriptional insulator element or a type of cis-regulatory element, it blocks enhancer-promoter communication to influence expression of genes^22^. Therefore, mutations in *CTCF* can lead to invasive cancers in breast, kidney (Wilm’s tumor) or prostate^23^. A previous study shows that CTCF epigenetically regulates p53 by codifying an open chromatin conformation that shields the p53 gene promoter from repressive histone marks^24^. This provides evidence for the critical role CTCF plays in regulating the expression of tumor suppressor genes.

In this study, we isolated two liver stem cell lines (HepCY & HepCO) from normal young (CY) and old human (CO) liver tissues and determined TP73 expression in normal human liver stem cells, hepatocellular carcinoma (HCC) cell lines (HepG2, SNU398, SNU449 & SNU475), gastrointestinal (GI) cancer cell lines (Caco2 & HCT116) and normal skin fibroblasts cell line (HS27) to demonstrate the correlation of TP73 expression in tumorigenesis. We also studied the effect of DNA methylation on the expression TP73 in various neoplastic tissues and cancer cell lines.

## Results

### Normal hepatocyte stem-like cell culture and characterization

**Figure 1:**
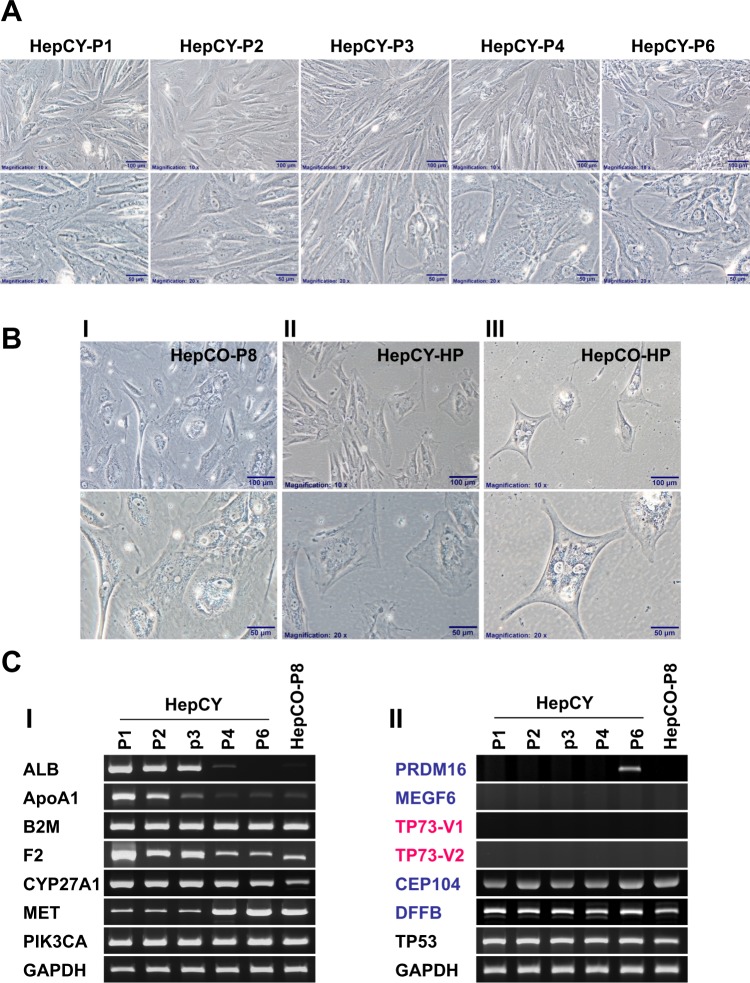
Morphological and Functional Characterization of Human Hepatocyte stem-like cells, HepCY and HepCO. (**A**) Photomicrographs showing phase contrast microscopy of morphological studies of HepCY from passage 1 to passage 6, Objective magnification: 10X upper panel (Scale Bar: 100 µm), 20X lower panel (Scale Bar: 50 µm). (**B**) Phase-contrast photomicrographs showing morphologic changes of passage 8 HepCO (I) and high passage HepCY (II) & HepCO (III) toward the hepatocyte phenotype, Objective magnification: 10X upper panel (Scale Bar: 100 µm), 20X lower panel (Scale Bar: 50 µm); (**C**) The level of gene expression in different passages, HepCY cells and passage 8 HepCO cells determined by RT-PCR: (I) Hepatocyte-specific & related protein, (II) The expression levels of Tumor protein *TP53*, *TP73* and *PRDM16*, *MEGF6*, *CEP104 and DFFB* genes proximal to the TP73 gene locus in chromosome1p36.32 region. *Note: The row of bands representing the expression of each gene and separated by white spaces as shown in the gels displayed in panel C-I and C-II are cropped from full-length gels of the corresponding genes*. *The same exposures were made for each gel*. *The original gels for each figure are shown in the Supplementary Information File*.

Human hepatocyte stem-like cells, HepCY and HepCO, were generated from human primary hepatocytes and were cultured for two weeks. The resulting HepCY and HepCO colonies were passaged at 70–80% confluency within 7–10 days. The phase-contrast photomicrographs showed HepCY morphologic changes from atypical fibroblast-like cells to atypical epithelial-like cells (Fig. 1A) through passages 1 to 6. The morphology of HepCO in Passage 8 shows hepatocyte-like cell structure (Fig. 1B, panel I). Both cells of HepCY and HepCO in high passages (HP, over passage 10) start to grow slowly but not proliferatively with typical hepatocyte-like morphology (Fig. 1B panels II & III).

Considering that liver-specific protein expression can be detected in hepatocyte cells, we measured expression of four genes specific to liver cells: ALB (encoding albumin), APOA1 (encoding apolipoprotein A1), B2M (encoding beta-2-microglobulin), F2 (encoding thrombin), and three genes that are highly expressed in liver cells: CYP27A1(encoding Cytochrome P450 family 27 subfamily A member 1) as well as a proto-oncogene MET (encoding met proto-oncogene, receptor tyrosine kinase) and a oncogenic gene PIK3CA (encoding phosphatidylinositol-4,5-bisphosphate 3-kinase catalytic subunit alpha). The results show that the level of gene expression of ALB, APOA1, F2 were dramatically down-regulated in high passages of HepCY and HepCO cells; the expression of CYP27A1 gene was slightly decreased in high passages and the levels of B2M and PIK3CA were not altered in different passages. In contrast, a dramatically elevated expression of MET gene occurs in passage 4 (Fig. 1C, panel I). Moreover, we determined alterations in the gene expression levels of tumor proteins, *TP53*, *TP73* and *PRDM16* (PR/SET domain 16), *MEGF6* (Multiple EGF like domains 6), *CEP104* (Centrosomal protein 104) *and DFFB* (DNA fragmentation factor subunit beta), which are in proximity to the *TP73* gene locus in chromosome 1p36.32 region. The results demonstrate the absence of visible alterations in expression of the genes among the different passages except for the *PRDM16* gene in passage 6 of HepCY (Fig. 1C, panel II).

These data indicate that HepCY and HepCO that are isolated from normal young and old human liver hepatocytes are liver *stem cells*, perhaps hepatocyte stem cells that can vouch for the tumor protein TP73’s lack of involvement in liver stem cell proliferation.

### TP73 gene expression in HCC and GI Cancer

**Figure 2:**
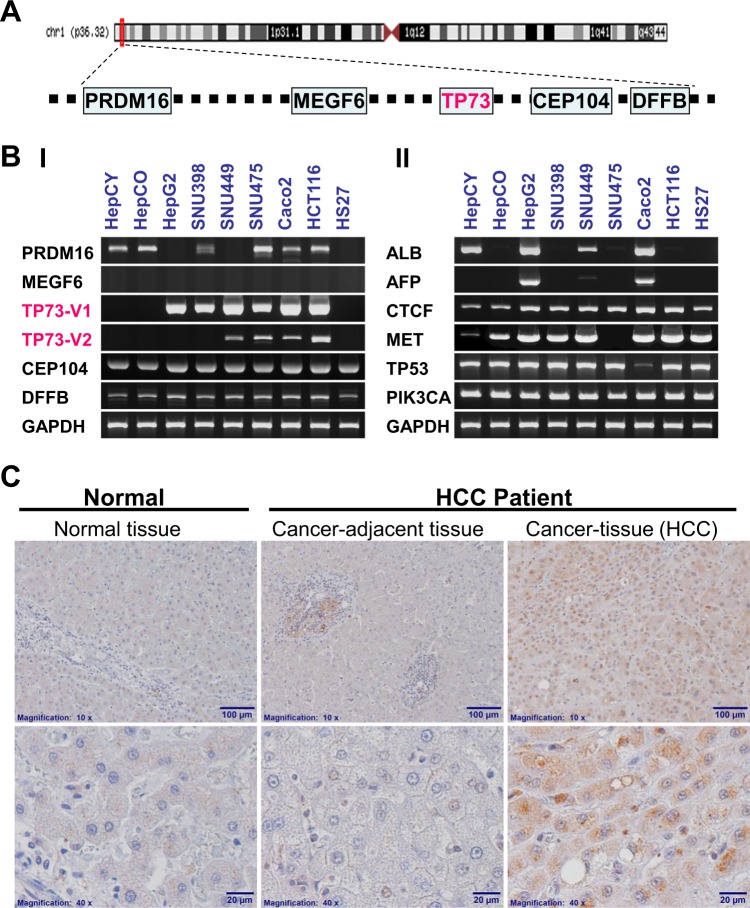
RT-PCR and Immunohistochemistry analyses of expression of TP73 and related-genes in human cell lines and tissues. **(A**) A schematic representation of several genes located in chromosome 1p36.32.; (**B)** Expression of TP73 gene and related-genes in normal human liver stem cells (HepCY & HepCO), HCC & GI cancer cell lines and normal human skin fibroblasts cell line (HS27): (Panel I) Tumor protein *TP73* and *PRDM16*, *MEGF6*, *CEP104 and DFFB* expression level close to the *TP73* gene located in chromosome 1p36.32 region, (II) related genes; (**C**) Immunohistochemistry analyses of TP73 expression in normal and HCC patients’ Cancer-adjacent tissue and Cancer tissues objective magnification: 10X upper panel (Scale Bar: 100 µm) and 40× lower panel (Scale Bar: 20 µm), three specimens were detected for each. This experiment was repeated three times with three different tissues to confirm the results. *Note: The row of bands representing the expression of each gene and separated by white spaces as shown in the gels displayed in B-I and B-II are cropped from full-length gels of the corresponding genes*. *The same exposures were made for each gel*. *The original gels for each figure are shown in the Supplementary Information File*.

The genes that are proximal to *TP73* (i.e. *PRDM16*, *MEGF6*, *CEP104 and DFFB*) in the region of chromosome 1 p36.32 (Fig. 2A), were assessed for their expression in human normal liver stem cells (HepCY and HepCO), hepatocellular cancer (HCC) cell lines (HepG2, SNU398, SNU449 & SNU475), gastrointestinal cancer (GI) cell lines (Caco2 & HCT116), and normal skin fibroblast cell line (HS27) by RT-PCR (Fig. 2BI). Surprisingly, the results show that TP73 only expresses in cancer cell lines albeit to varying levels but not in normal liver stem cells (HepCY and HepCO) or in normal foreskin human fibroblast cell line (HS27). We further analyzed related genes (*ALB, AFP, CTCF, MET, TP53* and *PIK3CA*) in human normal liver stem, hepatocellular cancer (HCC) cell lines, gastrointestinal cancer (GI) cell lines and normal skin fibroblasts cell line by using RT-PCR (Fig. 2BII). The results show that these genes may not be related to tumorigenesis. The expression of tumor protein p73 (TP73) in cancer cells suggests that it is positively correlated with tumorigenesis.

To confirm the elevated expression of *TP73* gene in tumorigenesis, we analyzed the TP73 protein level in human normal liver tissues and liver non-cancerous (Cancer-adjacent tissue) and cancerous tissues of HCC patients by immunohistochemistry. The results show that TP73 only expresses in HCC patients (Fig. 2C).

### *TP73* gene expression is activated at its promoter site by DNA methylation

**Figure 3:**
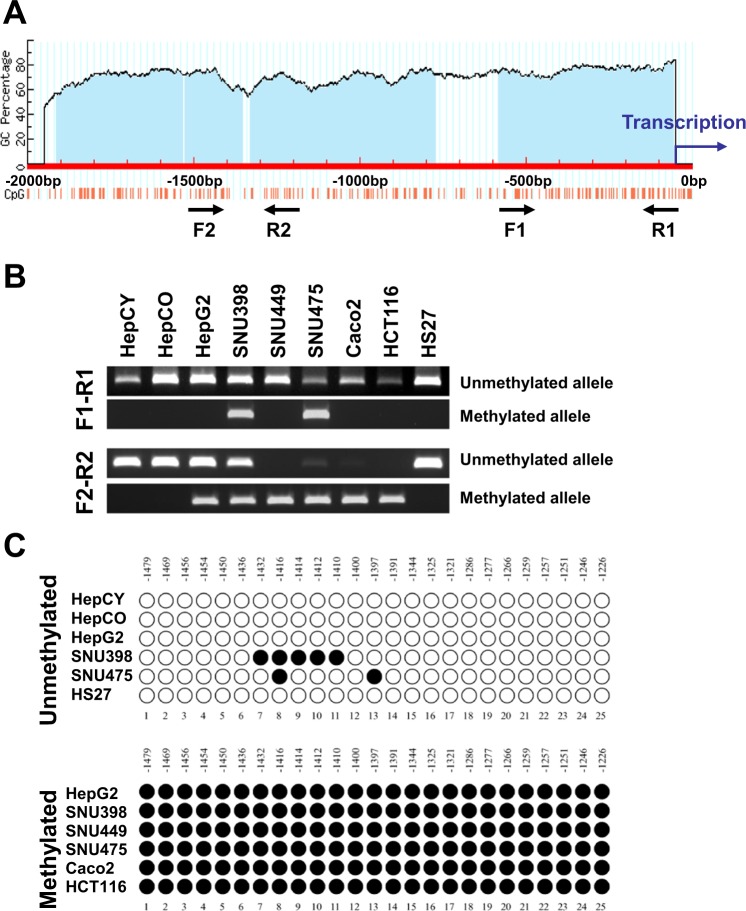
DNA methylation pattern of *TP73* gene promoter in normal human liver stem cells, HCC&GI cancer cell lines and normal human skin fibroblasts cell line. (**A)** Schematic outline for the sequence of *TP73* promoter and CpG islands. **(B)** Methylation status of *TP73* promoter in normal human liver stem cells (HepCY & HepCO), HCC & GI cancer cell lines and normal human skin fibroblasts cell line (HS27) detected by MSPCR **(C)** DNA methylation pattern of *TP73* gene promoter in normal human liver stem cells (HepCY & HepCO), HCC & GI cancer cell lines and normal human skin fibroblasts cell line (HS27) identified by bisulfite sequencing. *Note: The row of bands representing the expression of each gene and separated by white spaces as shown in the gels displayed in panel B are cropped from full-length gels of the corresponding genes*. *The same exposures were made for each gel*. *The original gels for each figure are shown in the Supplementary Information File*.

DNA methylation patterns are often altered significantly in cancer cells including those from HCC patients. Growing evidence suggests that aberrant DNA methylation of CpG islands around promoter regions can have the same effect as coding region mutations, leading to the inactivation of tumor suppressor genes^18^. Because the promoter region of TP73 contains four typical CpG islands (Fig. 3A), we examined their methylation state in genomic DNA isolated from nine cell lines (three normal cells and six HCC & GI cancer cell lines) utilizing methylation-specific PCR (Fig. 3B) and bisulfite sequencing (Fig. 3C). These results showed a positive correlation between high-levels of TP73 expression and methylation upstream of the TP73 promoter in human HCC and GI cancer cell lines (Fig. 2). In six HCC and GI cancer cell lines, we observed dramatically markedly expression of TP73, cytosine residues of CpG dinucleotides in the TP73 promoter region (−1479 to −1226), which were almost completely methylated, whereas those cytosine residues in normal cell lines (HepCY, HepCO and HS27), which lost TP73 expression, were entirely methylation-free (Fig. 3C). Thus, our results confirmed hypermethylation in this region of the TP73 promoter, which activates TP73 expression. The data demonstrate a comprehensive profile of TP73 activation at its promoter site by DNA methylation in human HCC cell lines as well as GI Cancer cell lines.

### CTCF and TP53 are involved the regulation of *TP73* gene expression

**Figure 4:**
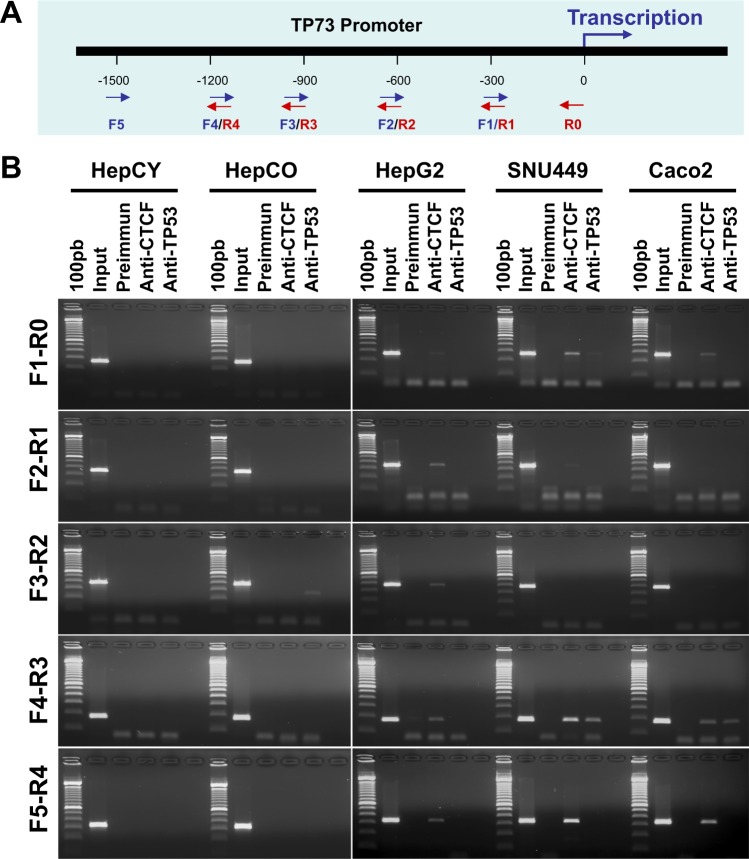
Role of TP53 and CTCF in regulation of TP73 gene expression. (**A**) Ideogram representing primers used in this ChIP assay for TP73 promoter. (**B**) Regulation of TP73 gene expression in normal human liver stem cells (HepCY & HepCO) and HCC&GI cancer cell lines by ChIP assay. These results were produced from triplicate experiments. *Note: The row of bands representing the expression of each gene and separated by white spaces as shown in the gels displayed in panel B are cropped from full-length gels of the corresponding genes*. *The same exposures were made for each gel*. *The original gels for each figure are shown in the Supplementary Information File*.

**Figure 5:**
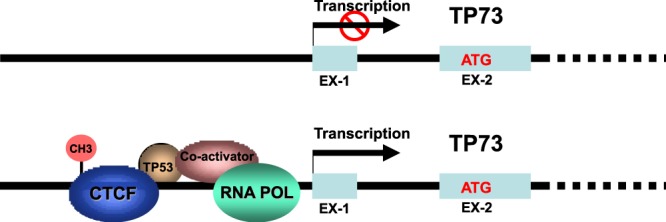
Schematic representation of presumable mechanism of regulation of TP73 gene expression.

CTCF is a chromosomal networking protein CCCTC binding factor and a key regulator and repressor of IGF2^21^. CTCF, as a transcriptional insulator element, can block communication between enhancers and upstream promoters, thereby regulating expression^23^. We further investigated the regulation of *TP73* gene expression by ChIP assay (Fig. 4A). Results show that TP53 association with CTCF involved *TP73* gene regulation by binding to TP73 promoter (Fig. 4B) in hepatocellular cancer (HCC) cell lines (HepG2 & SNU449) and gastrointestinal cancer (GI) cell line (Caco2). When compared to normal liver hepatocyte stem-like cells and normal skin fibroblasts, CHIP assay demonstrates the dysregulation of TP73 expression in HCC cells and GI cancer cells by TP53 and CTCF, possibly due to hypermethylation of TP73 promoter region (−1479 to −1226). This shows that CTCF regulates human *TP73* gene expression through direct interaction with its homologue protein, TP53. However, when the TP73 promoter is methylated as shown in cancer cells, the dual CTCF-TP53 regulation is blocked. A schematic representation of presumable mechanism of regulation of TP73 gene expression is shows in Fig. 5.

## Discussion

Cancer is the result of uncontrolled cell proliferation due to genetic DNA mutation or epigenetic DNA methylation among other possible etiologies. Self-renewal is a property shared by both cancer and normal cells^25,26^. During the past several decades, most studies have used the paired normal (non-proliferating cells) and the tumor tissues (proliferating cells) for studying the gene expression patterns in tumorigenesis. To study gene expression involving cell proliferation and differentiation and various underlying mechanisms for such possibilities and key features in tumorigenesis, the best approach is to compare normal stem cells with cancer cells or cancer stem cells^27^. Our data in this study showed that tumor protein TP73 expression is exclusive to cancer cells (Fig. 2B-I). By comparison, normal proliferating liver stem cells (HepCO & HepCY) and normal proliferating fibroblasts cell line (HS27), clearly show that TP73 is not involved in cell proliferation but is only involved in cell tumorigenesis. In other words, the function of TP73 protein is not required for cell proliferation per se, but it is related to cell tumorigenesis^28^. Moreover, by determining the *CTCF, MET, TP53* and *PIK3CA* gene expression levels in normal proliferating liver stem cells (HepCO & HepCY) and normal proliferating fibroblasts cell (HS27) (Fig. 2B-II), our results also indicated that these genes in their normal status are not involved in promoting cell tumorigenesis.

Epigenetics involves heritable modification of gene expression rather than alteration of the genetic code itself. However, collaborations may exist between epigenetic changes which occur in all human cancers and genetic alterations which occur at the base of the DNA to drive the cancer phenotype. Epigenetic changes are not limited to only DNA methylation or histone modifiers. These changes may also occur with chromatin remodelers, microRNAs, and other apparatuses of chromatin^16,18^. DNA methylation, however, is the primary driver of transcriptional silencing, a hallmark of cancer cells^18,29,30^. Our results in the DNA methylation study showed that hypermethylation within the TP73 promoter activates *TP73* gene expression in cancer cells (Figs. 2 and 3). Most DNA methylation sites control gene expression and therefore involve promoters that are associated with CpG islands. For instance, Gomez *et al*., observed that the methylation of varied CpG islands of the TP73 promoter differed significantly within its molecular subtypes in that he *TP73* gene was not expressed when its promoter was methylated^28^. Their study further showed that there was a higher expression of exon 3′ of p73 (expressed only in ΔNp73 isoform reflecting a high histologic grade) in patients with wild-type p53. Previously, we showed that DLL4, a notch ligand, is silenced by DNA methylation at its promoter site^31^. Interestingly, it is rare to find DNA methylation at promoter sites in correlation with gene activation in tumorigenesis. We know of only one such example involving IGF2, in which a DNA methylation within the embossed IGF2–H19 locus’s differentially methylated region (DMR) activates IGF2 expression^32^. H19 DMR regulates the genomic imprinting of IGF2 and H19 genes by using both a non-methylated DMR on the maternal chromosome, which shields or insulates IGF2 from enhancers; and a methylated DMR on the paternal chromosome, which disables the adjacent H19. These mechanisms seem to reflect the interplay among CTCF, histone deacetylases and intact chromatin insulator complexes^33–35^. Currently, there are only 14 biomarkers derived from DNA-methylation studies that have practical and commercial applications for clinical tests in cancer diagnosis^36^. Our observations demonstrate that a positive correlation exists between TP73 expression and DNA methylation in its promoter regions in tumorigenesis. This finding may prove significant for the development of future diagnostic applications.

CTCF is a multifunctional protein with multiple roles. It acts as a transcriptional activator, a repressor or an insulator containing a highly conserved 11 zinc finger domains. It is a CCCTC-binding factor or chromosomal networking protein. It plays a critical role as a gene regulator and repressor of IGF2 when it mediates insulation at the *H19-Insulin-like growth factor* 2 (*Igf2*)^21^. As a transcriptional insulator, CTCF can interfere with the network of enhancers and upstream promoters, thereby regulating imprinted (parent-of-origin-specific) expression^21^. This key regulatory component of CTCF and the critical role methylation plays in controlling this locus was shown by methylation of the CTCF-binding site at this locus, which resulted in blockage of the binding of the zinc finger protein^33,35^. Therefore, mutations in the *CTCF* gene can have far-reaching effects on initiation and development of cancer. Published reports have linked CTCF mutation to invasive cancers of the breast and prostate as well as Wilms’ tumors^24^.

The TP53 signaling pathway is responsive to an array of cellular stresses that activate the TP53 protein, which regulates the expression of downstream genes that target cell cycle arrest, apoptosis, DNA repair, senescence, etc. Loss of TP53 function, through mutations in TP53 itself or perturbations in pathways signaling to TP53, is a common feature in most human cancers^36^. The most consequential role of p53 as a transcription factor and the “guardian of the genome” to integrate cellular responses by activating or repressing the expression of several target genes and microRNAs^37,38^. As for p53 epigenetic regulation, recent studies have shown that CTCF guards against p53 promoter repression by histone marks through the provision of an open chromatin configuration for p53^24^. This evidence provides support for the germane role of CTCF in the regulation of epigenetic effects of tumor suppressor genes and cancer development^24^. Likewise, our data confirm that TP53 and CTCF jointly and directly influence the activation of the TP73 promoter. Concomitantly, it can be concluded that CTCF is a potential activator and regulator of TP73 (Fig. 4) even though the mechanisms by which the CTCF/TP53 alliance is controlled by cellular signaling are not delineated. Based on our experimental findings, we propose a model or a scheme, which may exemplify the mechanism of this tightly regulated partnership (Fig. 5).

In summary, identifying the molecular mechanisms underlying these methylation changes will require a detailed understanding of gene regulation and chromatin remodeling that might shed light on cancer initiation and progression. In the context of this research, we observed a close association among CTCF, TP53 and DNA methylation at the TP73 promoter site. Further work on defining the setting of TP73 expression under cellular and pathological conditions will be pivotal for designing and implementing effective therapeutic regimen for cancer.

## Methods

### Cell culture

The cell lines and human tissues employed in this study have been approved by the Institutional Review Boards (IRBs) of Howard University and Georgetown-Howard Universities Center for Clinical and Translational Science (GHUCCTS). Adult patients whose tissues were processed at MedStar Georgetown University Hospital and used for the immunohistochemistry procedure signed informed consent according to IRB guidelines. The tissues were collected at the time of surgery and immediately stored in liquid nitrogen or −80 °C freezer until use. These liver tissues were supplied by the Lombardi Comprehensive Cancer Center Histopathology & Tissue Shared Resources. All methods and experimental protocols were performed according to the pertinent guidelines and regulations approved by Howard University as well as GHUCCTS’ IRBs. HCC diagnosis was based on imaging criteria or clinical stages; whereas histology was based on the TNM system.

Human hepatocyte stem-like cells, HepCY and HepCO, were generated from human primary hepatocytes (GIBCO, Cat# HMCPTS). The original human primary hepatocytes from GIBCO were grown in DMEM media containing 12.5% fetal bovine serum (FBS) and 1X NEAA at 37 °C, and 5% CO_2_ (Life Technologies, Bethesda, MD). After 2 weeks of culture, colonies were observed with an Olympus IX73 light microscope equipped with a phase contrast apparatus. The cells were split every 7–10 days at 70–80% confluency. The HepCY human hepatocyte stem-like cells were generated from a 21-year old human primary hepatocyte; whereas HepCO cells were generated from a 60-year old human primary hepatocyte. HCC (HepG2, SNU3958, SNU449, and SNU475) and GI (Caco2 and HCT116) cancer cell lines, and normal human skin fibroblasts cell line (HS27) were obtained from ATCC (Manassas, VA) and cultured following the instructions of the supplier. Cells underwent low passages and were harvested at 75–90% confluency.

### RT-PCR

The primers used for TP73 transcript variant 1 amplifications were as follows: forward/5′-GGA AGA TGG CCC AGT CCA CCG -3′ reverse/5′-GTG GAT CTC GGC CTC CGT GAA C-3′. The primers used for TP73 transcript variant 2 amplifications were as follows: forward/5′-ACC ATG CTG TAC GTC GGT GAC CC-3′ reverse/5′-GTG GAT CTC GGC CTC CGT GAA C-3′. The primers used in RT-PCR for all other mRNA in this study were: forward primer from start codon around 24 bp; reverse primer from stop codon around 24 bp; Tm around 60 °C and amplification for full length mRNA. All other primers were as follows: ALB forward/5′-ATG AAG TGG GTA ACC TTT ATT TCC CTT CTT T-3′ reverse/5′-TTA TAA GCC TAA GGC AGC TTG ACT TGC AG-3′; APOA1 transcript variant 1 forward/5′-AGG ATG AAA GCT GCG GTG CTG-3′ reverse/5′-CTG GGT GTT GAG CTT CTT AG TGT ACT CC-3′; B2M forward/5′-ATG TCT CGC TCC GTG GCC TTA-3′ reverse/5′-GCT GCT TAC ATG TCT CGA TCC CAC TTA AC-3′; F2 (Thrombin) transcript variant 1 forward/5′-ACA CTA TGG CGC ACG TCC GAG-3′ reverse/5′-CCC TAC TCT CCA AAC TGA TCA ATG ACC-3′; CYP27A1 forward/5′-ATG GCT GCG CTG GGC TGC-3′ reverse/5′-TCA GCA CTG TCT CTG CAG GAA CTG C-3′; MET transcript variant 1 forward/5′-ATA ATG AAG GCC CCC GCT GTG C-3′ reverse/5′-CTA TGA TGT CTC CCA GAA GGA GGC TG-3′; PIK3CA forward/5′-ACA ATG CCT CCA CGA CCA TCA TCA-3′ reverse/5′-GTT CAA TGC ATG CTG TTT AAT TGT GTG G-3′; PRDM16 transcript variant 1 forward/5′-ACC ATG CGA TCC AAG GCG AGG-3′ reverse/5′-GAG GTG GTT GAT GGG GTG AAA TGC-3′; MEGF6 forward/5′-ACG ATG TCG TTC CTT GAA GAG GCG-3′ reverse/5′-GTG CCT CGC TGG TCC ACC GCT-3′; CEP104 forward/5′-AGA ATG CCC CAC AAG ATT GGA TTT GTA G-3′ reverse/5′-GCG CTT GGC GTA CGT CCT GCT-3′; DFFB transcript variant 1 forward/5′-GCA ATG CTC CAG AAG CCC AAG AG-3′ reverse/5′-CTG GCG TTT CCG CAC AGG CTG-3′; TP53 transcript variant 1 forward/5′-GCC ATG GAG GAG CCG CAG TCA-3′ reverse/5′-TCA GTC TGA GTC AGG CCC TTC TGT CTT-3′; GAPDH transcript variant 1 forward/5′-ATG GGG AAG GTG AAG GTC GGA GTC-3′ reverse/5′-TAC TCC TTG GAG GCC ATG TGG GC-3′;

### Immunohistology (IH)

Immunohistochemical staining was performed by the Lombardi Comprehensive Cancer Center Histopathology & Tissue Shared Resources at Georgetown University Medical Center. Briefly, immunohistochemical staining of normal and tumor tissue samples of liver was performed for human TP73 made in rabbit. Five-micron sections from formalin fixed paraffin embedded (FFPE) tissues were de-paraffinized with xylenes and rehydrated through a graded alcohol series. Heat induced epitope retrieval (HIER) was performed by immersing the tissue sections at 98 °C for 20 minutes in 10 mM citrate buffer (pH 6.0) with 0.05% Tween. Immunohistochemical staining was performed using a horseradish peroxidase labeled polymer #K4003 (Dako North America, Carpinteria, CA) according to manufacturer’s instructions. Briefly, slides were treated with 3% hydrogen peroxide and 10% normal goat serum for 10 minutes each and exposed to primary antibody TP73 (1:60, Abcam, Cat # ab14430) diluted in 1X TBS with 0.05% Tween 20 (Fisher, Pittsburg, PA) overnight at 4 °C. Slides were exposed to the HRP labeled polymer for 30 min and DAB chromagen (Dako) for 5 minutes. Slides were counterstained with Hematoxylin (Fisher, Harris Modified Hematoxylin), blued in 1% ammonium hydroxide, dehydrated, and mounted with Acrymount. Consecutive sections without the primary antibody were used as negative controls. The wash buffer used was 1X TBS with 0.05% Tween 20 (Fisher).

#### DNA methylation analysis

Genomic DNA was bisulfite-modified with an EpiTect Bisulfite Kit (Qiagen, CA, USA) according to the manufacturer’s protocols. Prediction of CpG islands in TP73 promoter and primer design for methylation-specific PCR was possible through the use of a web software (www.urogene.org); Primer pairs used for HepCY, HepCO, HepG2, SNU449, Caco2, HCT116 and HS27 cell lines in methylation-specific PCR at TP73 promoter down stream (F1/R1) were methylated forward/5′-GCG GCG GTT AGG AGA GAT TCG-3′ reverse/5′-CTA CAA CCG TCG CAA CCC CG-3′ and unmethylated forward 5′-TAG TGG TGG TGG TTA GGA GAG ATT TGG-3′ reverse/5′-CCT ACC TAC AAC CAT CAC AAC CCC A-3′; Primer pairs used for methylation-specific PCR and bisulfite sequencing at TP73 promoter upstream (F2/R2) were methylated forward/5′-CGT TTA GTT TCG GGT TTG TTT TTC GC-3′ reverse/5′-CGC AAA CTA AAT TCT CTA ACC GCA ACG-3′ and unmethylated forward 5′-GGG TGT TTA GTT TTG GGT TTG TTT TTT GT-3′ reverse/5′-GTG GGT GAG TTA TGA AGA TGT GTG AGT TAG TT-3′.

### Chromatin-immunoprecipitation (ChIP) assays

ChIP assay was performed using a ChIP assay kit according to manufacturer’s instructions (Upstate Biotechnology). Rabbit anti-TP53 (Cat#2527) and Rabbit anti-CTCF (Cat#3418) for ChIP assay were from Cell Signaling Technology (Boston, MA). Primers for TP73 promoter were as follows: forward/F1) 5′-GAC CTG CTT CGG CCC TGC GT-3′; F2) 5′-CAG GAG AAG TGG GTG GCA AGC C-3′ F3) 5′-GGC CTG GTG TAC GTG GTC GAG-3′; F4) 5′-GGC TTC ACT GAC GCG ACT TTC CAA-3′; F5) 5′-CCA GGG TCC TGC TCG TAC CTC C-3′. reverse/R0) 5′-CGG GTT ATA TGG GCG CGG GGA G-3′; R1) 5′-ACG CAG GGC CGA AGC AGG TC-3′; R2) 5′-GGC TTG CCA CCC ACT TCT CCT G-3′; R3) 5′-CTC GAC CAC GTA CAC CAG GCC-3′; R4) 5′-TTG GAA AGT CGC GTC AGT GAA GCC-3′.

## Supplementary information

Supplementary Information

## Data Availability

Our data are available by request.
