# Supplementary material for: DNA Methylation Activates TP73 Expression in Hepatocellular Carcinoma and Gastrointestinal Cancer
Source: Sci Rep. 2019 Dec 18;9:19367. doi: 10.1038/s41598-019-55945-7 (PMC6920427; doi:10.1038/s41598-019-55945-7)
Supplement: Supplementary file 1 — Supplementary Information [file 41598_2019_55945_MOESM1_ESM.pdf]

DNA Methylation Activates TP73 Expression in Hepatocellular Carcinoma and Gastrointestinal Cancer

Zhixing Yao[1], Cristina Di Poto[2], Grace Mavodza[1,3], Everett Oliver[1,2], Habtom W. Ressom[2] and Zaki A. Sherif[1*]

[1]Department of Biochemistry & Molecular Biology, College of Medicine,

Howard University, Washington DC 20059

[2]Department of Oncology, Lombardi Cancer Center, Georgetown University,

Washington DC 20007

[3] Department of Pharmacology
Hershey College of Medicine, Pennsylvania State University, PA 17033

*To whom correspondence should be addressed:
Zaki A. Sherif, Department of Biochemistry & Molecular Biology, College of Medicine, Howard University, 520 W Street NW, Washington, DC 20059: Telephone (202)-806-3832: Fax: (202)-806-9757; E-mail: zaki.sherif@howard.edu

SUPPLEMENTARY FIGURES

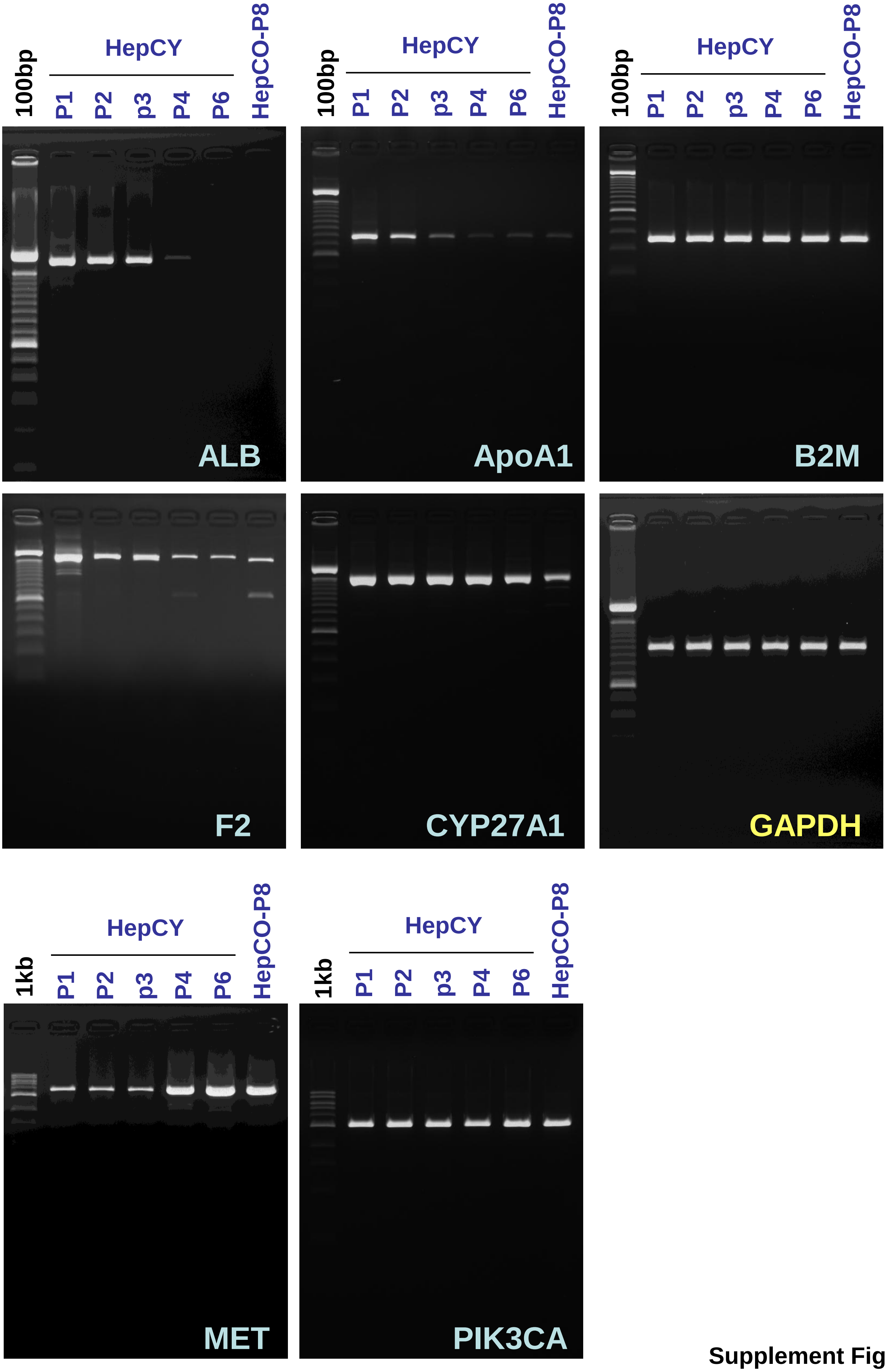

Supplement Figure 1

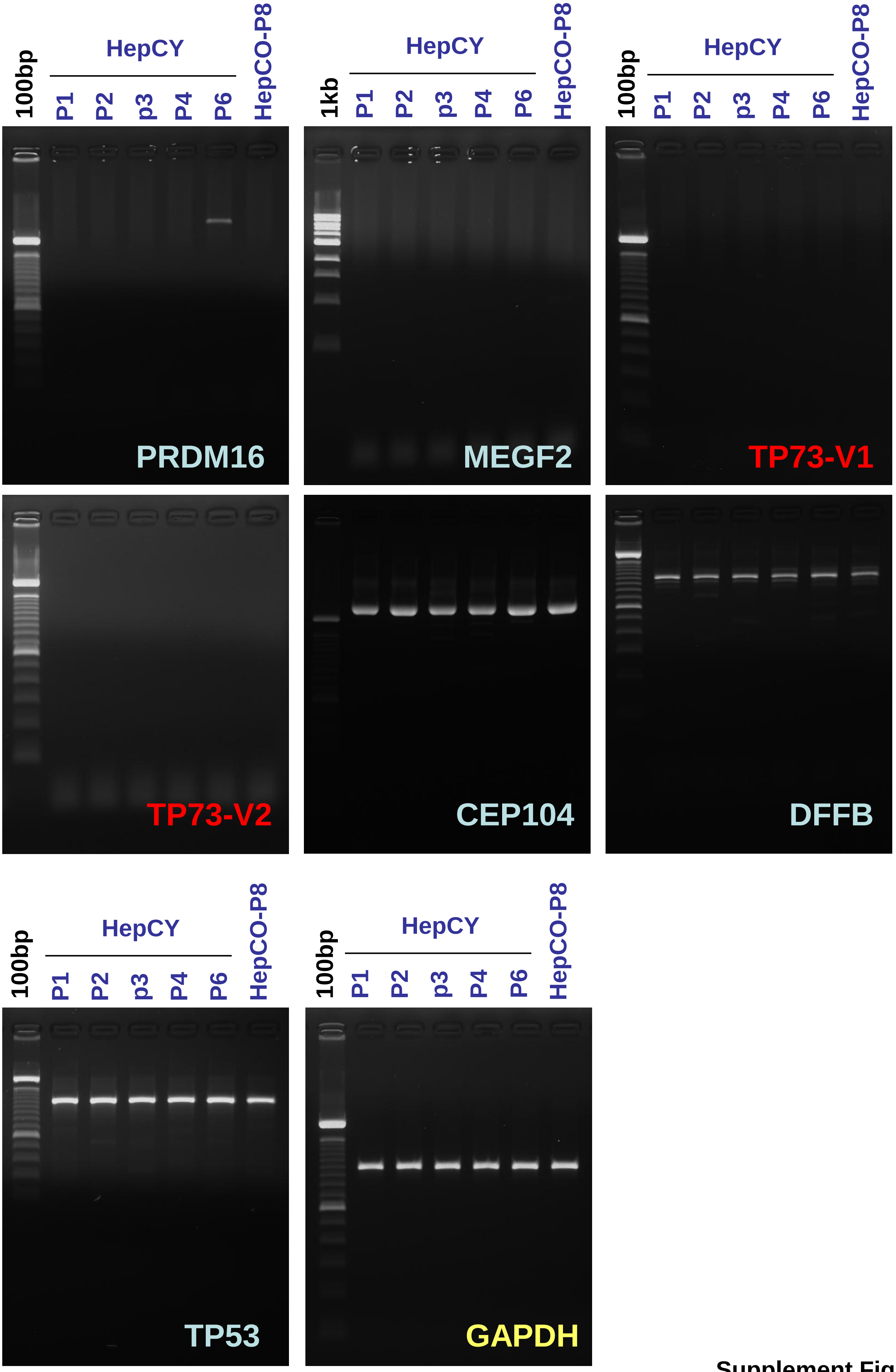

Supplement Figure 2

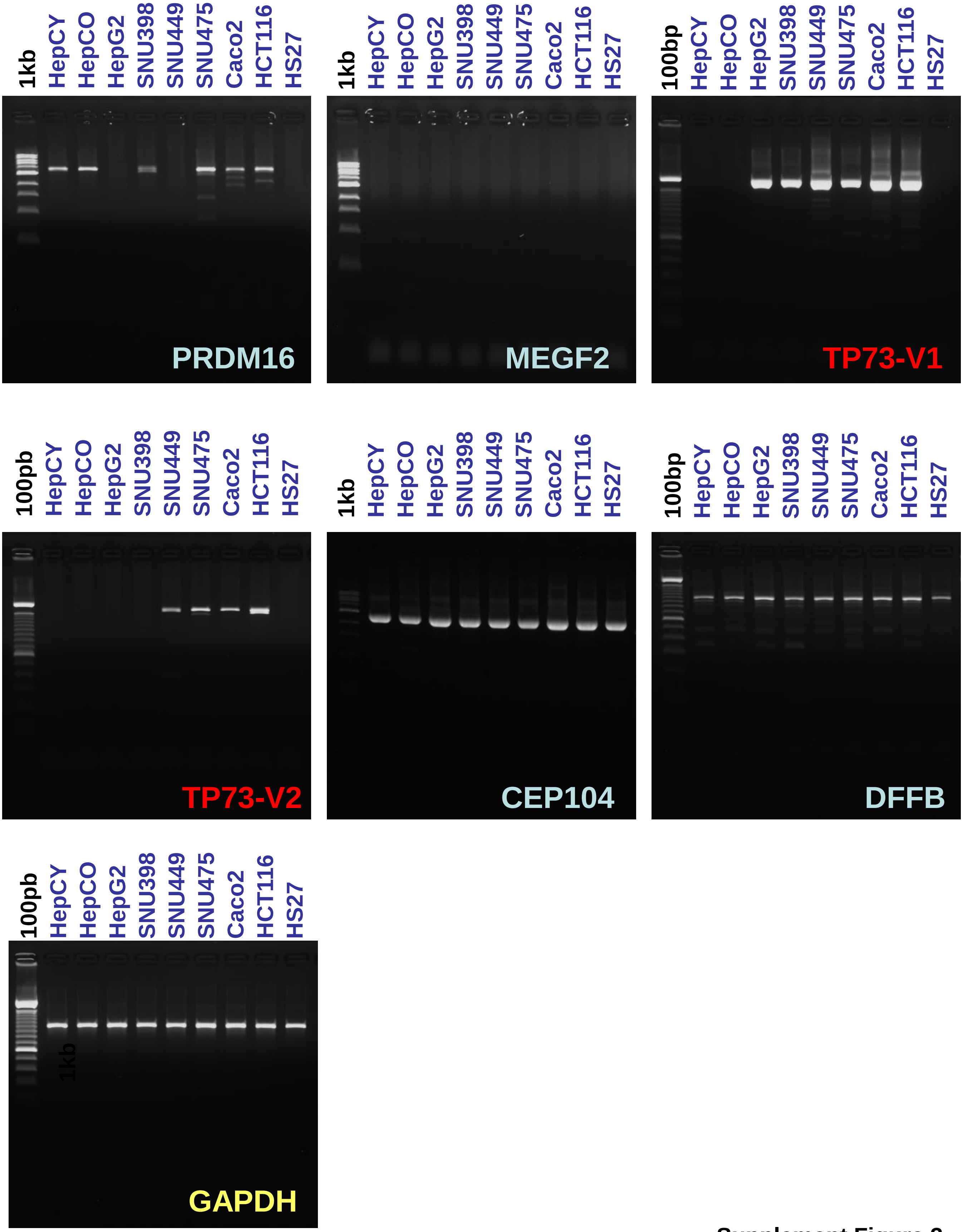

Supplement Figure 3

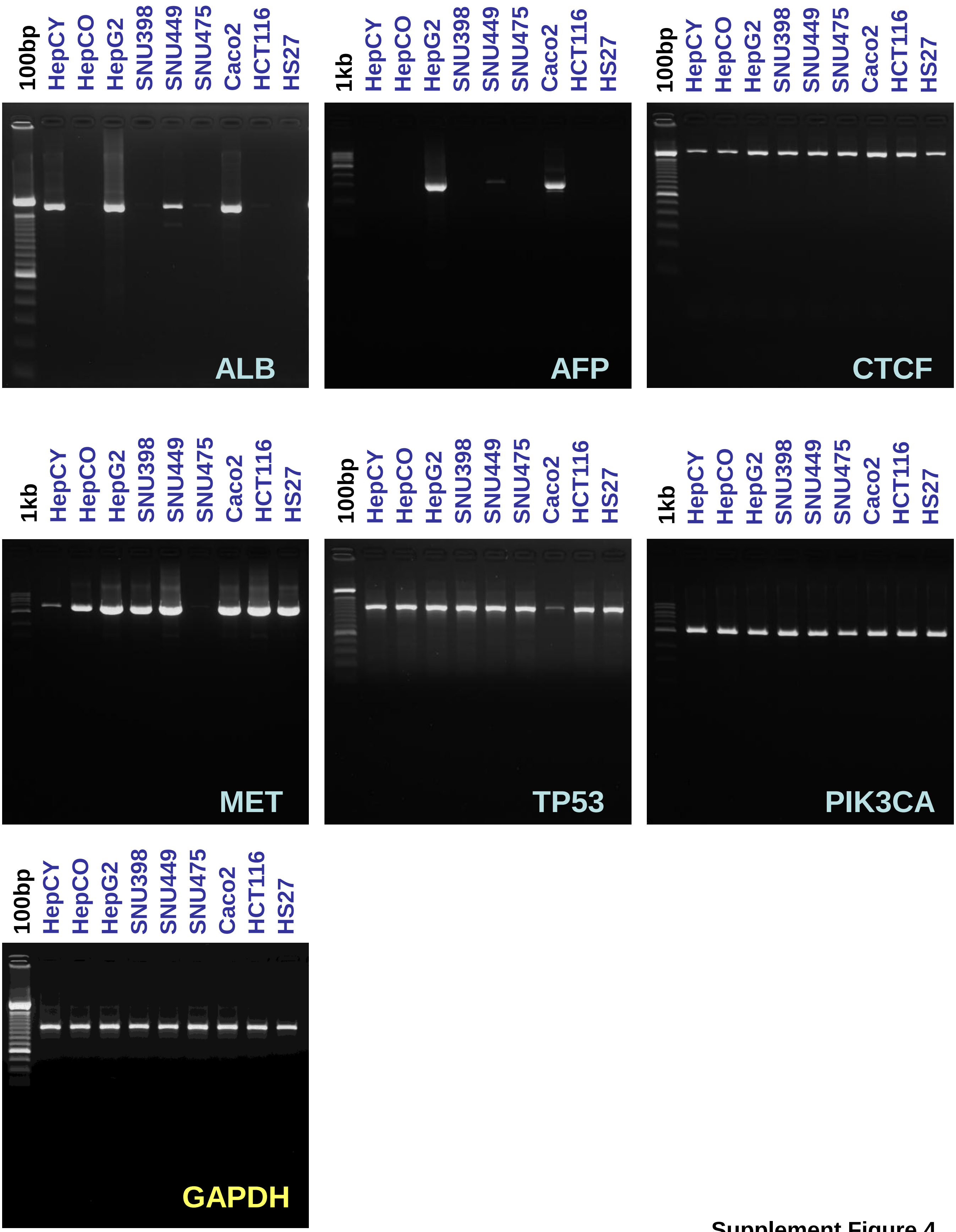

Supplement Figure 4

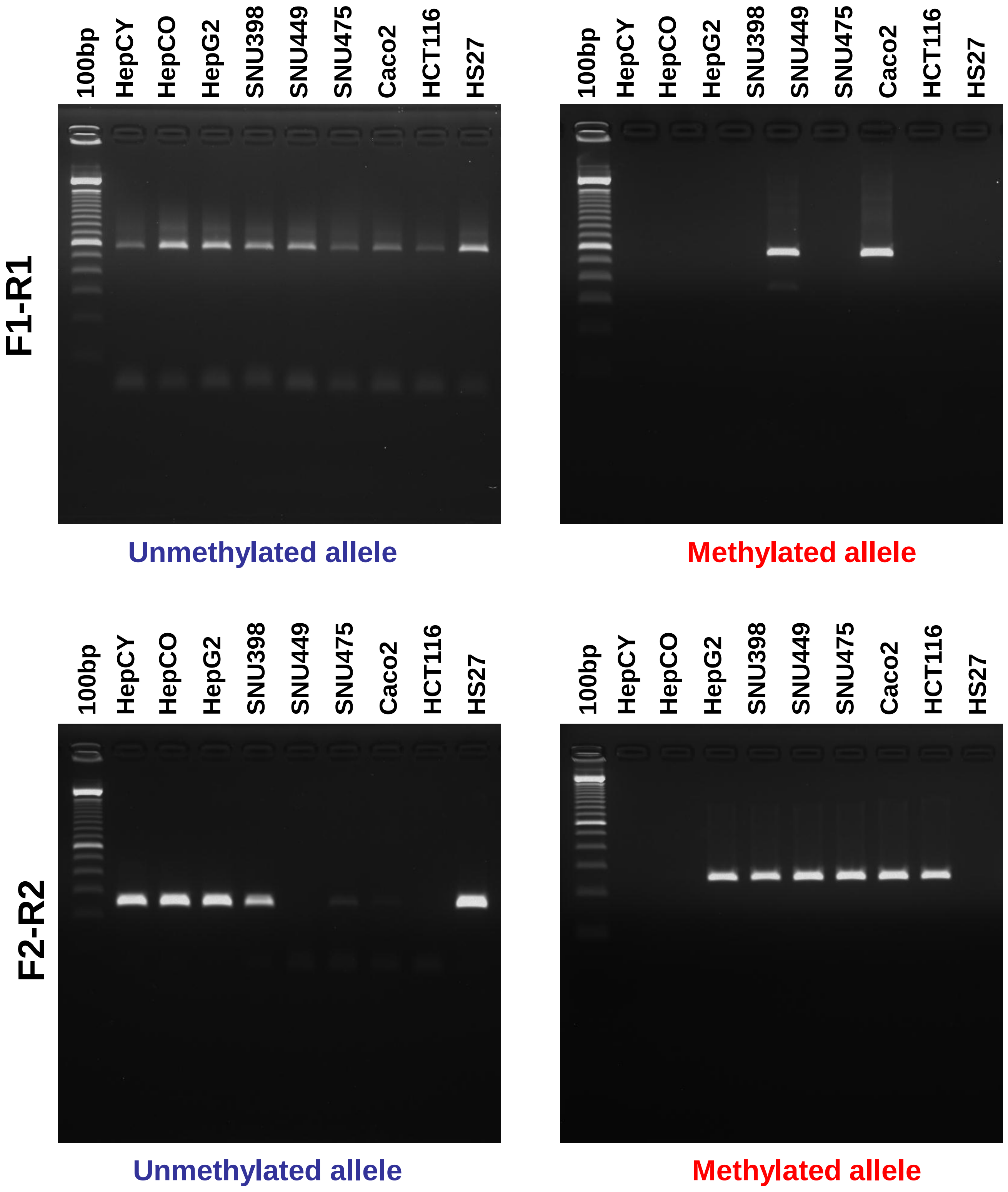

Supplement Figure 5
